# Regulatory network characterization of anthocyanin metabolites in purple sweetpotato *via* joint transcriptomics and metabolomics

**DOI:** 10.3389/fpls.2023.1030236

**Published:** 2023-02-09

**Authors:** Jiping Xiao, Xiaoyu Xu, Maoxing Li, Xiaojie Wu, Huachun Guo

**Affiliations:** Tuber-Root Crop Research Institute, College of Agronomy and Biotechnology, Yunnan Agricultural University, Kunming, Yunnan, China

**Keywords:** purple sweet potato, flesh color, anthocyanin, transcriptomics, metabolomics, gene

## Abstract

**Introduction:**

Sweet potato is an important staple food crop in the world and contains abundant secondary metabolites in its underground tuberous roots. The large accumulation of several categories of secondary metabolites result in colorful pigmentation of the roots. Anthocyanin, is a typical flavonoid compound present in purple sweet potatoes and it contributes to the antioxidant activity.

**Methods:**

In this study, we developed joint omics research via by combing the transcriptomic and metabolomic analysis to explore the molecular mechanisms underlying the anthocyanin biosynthesis in purple sweet potato. Four experimental materials with different pigmentation phenotypes, 1143-1 (white root flesh), HS (orange root flesh), Dianziganshu No.88 (DZ88, purple root flesh), and Dianziganshu No.54 (DZ54, dark purple root flesh) were comparably studied.

**Results and discussion:**

We identified 38 differentially accumulated pigment metabolites and 1214 differentially expressed genes from a total of 418 metabolites and 50893 genes detected. There were 14 kinds of anthocyanin detected in DZ88 and DZ54, with glycosylated cyanidin and peonidin as the major components. The significantly enhanced expression levels of multiple structural genes involved in the central anthocyanin metabolic network, such as chalcone isomerase (CHI), flavanone 3-hydroxylase (F3H), dihydroflavonol 4-reductase (DFR), anthocyanidin synthase/leucocyanidin oxygenase (ANS), and glutathione S-transferase (GST) were manifested to be the primary reason why the purple sweet potatoes had a much higher accumulation of anthocyanin. Moreover, the competition or redistribution of the intermediate substrates (i.e. dihydrokaempferol and dihydroquercetin) between the downstream production of anthocyanin products and the flavonoid derivatization (i.e. quercetin and kaempferol) under the regulation of the flavonol synthesis (FLS) gene, might play a crucial role in the metabolite flux repartitioning, which further led to the discrepant pigmentary performances in the purple and non-purple materials. Furthermore, the substantial production of chlorogenic acid, another prominent high-value antioxidant, in DZ88 and DZ54 seemed to be an interrelated but independent pathway differentiated from the anthocyanin biosynthesis. Collectively, these data from the transcriptomic and metabolomic analysis of four kinds of sweet potatoes provide insight to understand the molecular mechanisms of the coloring mechanism in purple sweet potatoes.

## Introduction

Sweet potato [*Ipomoea batatas* (L.) Lam.] is viewed as the seventh largest staple crop in the world China is the current biggest producer accounting for 68% of the overall global sweet potato production (FAO (Food and Agriculture Organization), 2013). Four sweet potato cultivars are broadly cultivated in China and each contains distinct secondary nutrients that cause different flesh colors, such as white, orange, yellow, and purple (Tang et al., 2015). According to such metabolic differentiation, the subsequent consumption, processing, and development of sweet potato products are distinguished to a certain extent (Ruttarattanamongkol et al., 2016). For example, the orange flesh sweet potato enriched with trans-β-carotene (276.98 µg/g) could better satisfy the daily requirement of vitamin A for preschool children and, as a good source of provitamin A, could prevent night blindness (Low et al., 2007; Tumuhimbise et al., 2009; Mitra, 2012; US Agency for International Development, 2014). While the enrichment of flavonoids (i.e. anthocyanin) in purple flesh sweet potato has more applicability in the health-promoting (e.g. free-radical scavenging (Tsushida et al., 1994; Zhang et al., 2013), anti-cancer/inflammatory (Lim et al., 2013; Jiang et al., 2020) etc.), and industrial developments (e.g. raw material of natural pigment) (Sun et al., 2014; Wang et al., 2014; Zhi et al., 2019). In addition, since sweet potato is also an excellent source of carbohydrates and other nutrients, such as dietary fiber and minerals (Nurdjanah et al., 2022), it has been more recognized as a functional food source than the staple crop (Azeem et al., 2020).

In recent years, anthocyanin has attracted more and more attention due to its notable value as an important subgroup of the pigmental flavonoids that can be enormously accumulated in colorful tissues of plants (Hatier et al., 2008; Sun et al., 2018; Wang S. et al., 2016). Compared to other anthocyanin-rich plants like strawberry (*Fragaria×ananassa*), red cabbage (*Brassica oleracea*), and perilla (*Perilla frutescens*), the anthocyanins in purple sweet potato are found to be more promising for industrial usage. Specifically, since it is mainly synthesized from the 3,5-diglucoside derivatives of the cyanidin or peonidin, then mono/di-acylated with caffeic acid, ferulic acid, p-coumaric, and p-hydroxybenzoic acid to form a more color-stable structure attached with the glucose and sophorose glucosyl groups, it harbors better oxidative resistance (Kim et al., 2012; He et al., 2016; Wei et al., 2019). Besides, in terms of the anthocyanin content and composition, it was revealed that the anthocyanin accumulation in purple sweet potato had better performances in different germplasms. Hu et al. analyzed the total anthocyanin contents of 30 purple flesh sweet potato cultivars from Chongqing, China; a variable level range from 74.3 to 607 mg/100 g on a dry weight (DW) basis was reflected (Hu et al., 2016). Zhu et al. and Sun et al. reported that the major anthocyanin composition in purple sweet potato is either peonidin or cyaniding-based anthocyanin, suggesting that despite the anthocyanin biosynthesis in planta is generally conservative but it may still be differentiated in purple sweet potato (Zhu et al., 2010; Sun et al., 2018).

As a multi-enzymatic catalytic process, the flavonoid pathway is responsible for the production of anthocyanin, which is highly regulated by a suite of structural genes, such as dihydroflavonol 4-reductase (*DFR*), anthocyanidin synthase (*ANS*), UDP glucose-flavonoid 3-O-glcosyl-transferase (*UFGT*), and several transcription factors synergistically (Li et al., 2019). The crucial transcription factors (TF) controlling anthocyanin biosynthesis have been identified to be the *MYB*, *bHLH*, and *WD40* (Xie et al., 2012; Dixon et al., 2013). These TFs usually form a protein complex (MBW complex) to induce the expressions of the downstream structural genes (Xu et al., 2015; Bulgakov et al., 2017), of which the *IbMYB1* was found to be the key regulator in the storage roots of purple sweet potato, and that it either functions individually or can bound to the *bHLH* and *WD40* (Mano et al., 2007; Hichri et al., 2010; Li et al., 2016; Zhao et al., 2022). Hence, the elucidation of the plant anthocyanin biosynthesis as well as the relevant tissue-specific pigmentation is mostly concerned with the gene expressions and interactions in this respect (Li et al., 2019).

Yunnan province is famous for its abundant biodiversity. The promising collection of sweet potato germplasms in the province could therefore provide more opportunities to breed superior varieties that can better fit the ever-increasing social demand for healthy and functional food, for instance, the purple sweet potato (Meng and Guo, 2019). To support that, the given molecular mechanisms underlying the anthocyanin anabolism in the purple sweet potato root flesh, which is the primary economic organ for harvesting, should be investigated in-depth, particularly in the regional germplasms that have had more genetic variations relative to the existing cultivars (Mano et al., 2007; Liu et al., 2017). The known knowledge of other plants, such as Arabidopsis (Arabidopsis thaliana) (He et al., 2020), apple (Malus domestica)(Zhang et al., 2019), tomato (Solanum lycopersicum) (Sun et al., 2020), as well as the recently reported studies in purple sweet potato (Zhang et al., 2022; Zhao et al., 2022), would provide a solid reference foundation for relevant research.

In this study, a joint omics analysis combining transcriptomics and metabolomics that aimed to reveal the differences in the metabolic regulation of anthocyanin production in purple sweet potatoes was conducted. Four sweet potato materials including two purple sweet potato varieties named Dianziganshu No.88 (DZ88) and Dianziganshu No.54 (DZ54), which were recently developed by the Tuber-Root Crop Research Institute of Yunnan Agricultural University, and two non-purple sweet potato materials, 1143-1 (a self-bred white flesh line) and HS (a Chinese commercial cultivar with orange flesh), were selected for experimentation. By focusing on the central anthocyanin metabolic network acting on the root flesh pigmentation in the two unique Yunnan purple sweet potato germplasms DZ88 and DZ54, insights on the anthocyanin biogenesis in purple sweet potato as well as the production of high-value bioactive compounds have been revealed. Our results may provide useful information for genetic breeding improvements and functional food development for the purple sweet potato industry.

## Materials and methods

### Plant materials

**Figure 1:**
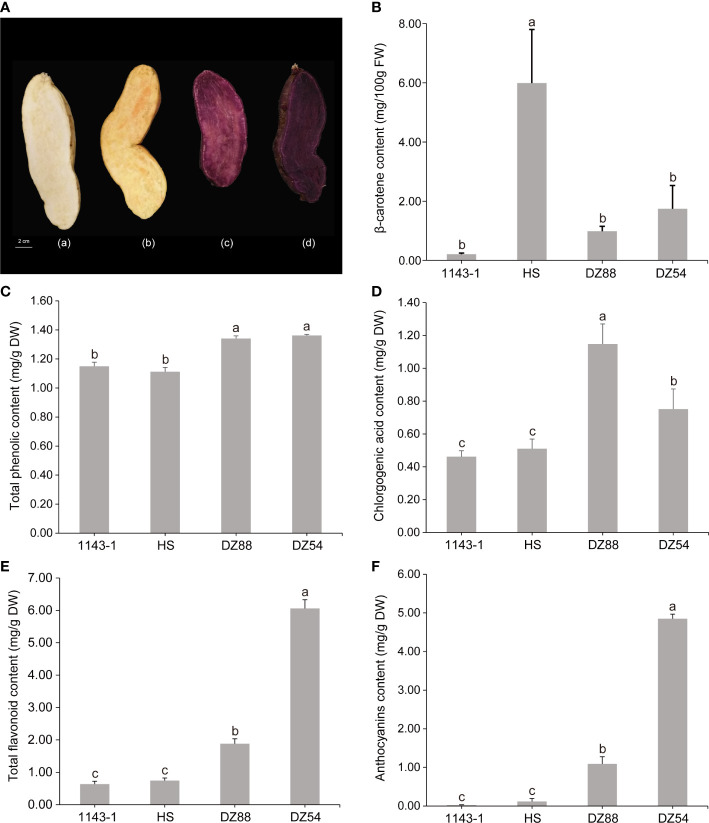
General phenotypes of the four experimental sweet potato materials. **(A)** Morphology and flesh color of the four sweet potato materials 1143-1 (white flesh), HS (orange flesh), DZ88 (purple flesh), and DZ54 (purple flesh), respectively. **(B)** Flesh β-carotene contents of the four sweet potato materials. **(C)** Flesh total phenolic contents (TPC) of the four sweet potato materials. **(D)** Flesh chlorogenic acid contents of the four sweet potato materials. **(E)** Flesh total flavonoid contents (TFC) of the four sweet potato materials. **(F)** Flesh anthocyanin contents of the four sweet potato materials. Error bars indicate standard errors (SEs) and the letters a, b, and c above each bar represent the significant difference according to the LSD (least significant difference) test.

Four sweet potato materials, including the white-fleshed (1143-1), orange-fleshed (HS), and two purple-fleshed (DZ88 and DZ54) lines were grown under the same conditions (23.5 °C/15.5 °C, N: 15 P: 5 K: 20). The mature roots of sweet potatoes were harvested at 120 days after the germination (**Figure 1A**). Six fresh roots of one variety were then immediately cut into tiny pieces and mixed into a duplicate, stored at −80°C for the subsequent RNA and metabolites extractions as well as other analyses. The process was repeated three times with each variety.

### Determination of β-carotene content

The β-carotene content was determined using the method of Zhang and Fang (2006). In summary, sweet potato tiny cubes (2.0g) were ground with a small amount of acetone and this was repeated 3-5 times until they became colorless. All the extracts and residues were collected in a 25mL volumetric flask, the volume was fixed with acetone to the scale, and centrifuged at 3000r/min for 5min. The supernatant was taken and the concentration of β-carotene was measured at 454nm with acetone as blank control by spectrophotometer (UV-1201, Shimadzu, Kyoto, Japan). The optical path was 1cm. The β-carotene content was calculated as the formula: y=13.1x-0.8 (te: 13.1 was the regression coefficient, x was the absorbance of acetone extract, and 0.8 was the linear regression intercept.)

### Determination of total phenolic content

The total phenolic content (TPC) in the mature roots of the four sweet potato materials was determined using the Folin–Ciocalteau assay method described by Waterhouse (Waterhouse, 2003). Absorbance was measured at a maximum wavelength of 760 nm, and TPC was recorded as micrograms of gallic acid (GA) (Sigma-Aldrich, St. Louis, MO, USA) per gram of the dry weight (μg GA/g DW). The standard curve for the TPC content calculation was prepared at five different concentrations of gallic acid ranging from 0.02 to 0.1 mg/mL(y=0.1708x–0.0025, R=0.9993).

### Determination of chlorogenic acid content

The chlorogenic acid content was measured by UV spectroscopy. Fresh sweet potato root samples were fully dried under - 80 °C and ground into fine powders, then moved into the 70% ethanol solution (1: 100, solid-liquid ratio) for the ultrasonic treatment for 10 min in order to isolate the polyphenols. The extraction solution was subsequently centrifuged at 5000 r for 10 min, followed by transferring 0.5 mL of the supernatant into 70% ethanol to volume 10mL. After that, the OD value was detected at 327 nm wavelength before the calculation of the content according to the standard curve (y = 63.44x-0.0043, R = 0.9997). The standard curve of the chlorogenic acid content was prepared *via* using the 0.05 mg/mL chlorogenic acid standard (Sigma-Aldrich, St. Louis MO, USA) dissolved in 70% ethanol (0 mL, 0.4 mL, 0.8 mL, 1.2 mL, 1.6 mL, 2 mL, 2.4 mL, 2.8 mL).

### Quantification of total flavonoid content

Total flavonoid content (TFC) was measured by sodium nitrite aluminum nitrate colorimetry following the method described in Do Q et al. (Do et al., 2014), but with some slight modifications. An extract sample with a 1% volume fraction of hydrochloric acid ethanol solution was placed in an ultrasonic water bath (30° C) for 30 min and then centrifuged for 30 min. After that, rutin was used as standard (Sangon Biotech, Shanghai, China) for quantification under 510 nm wavelength (y = 1.498x + 0.0079, R = 0.9994). The results were displayed as mg/g dry weight (DW).

### Determination of total anthocyanin content

The total anthocyanin content (TAC) was quantified using a spectrophotometric pH-differential method illustrated by Lee et al. (Lee et al., 2005) and Azeem M et al. (Azeem et al., 2020). TAC was presented as cyanidin-3-glucoside equivalents (mg/g DW).

### Quantitative real-time PCR analysis of anthocyanin biosynthetic structural genes and transcription factors expressions

Total RNA was prepared from the freshly sampled sweet potato roots using the RNA simple Total RNA Kit (Tiangen Biotech, Beijing, China), then the cDNA was obtained *via* the reverse transcription with the TransScript^®^-Uni One-Step gDNA Removal and cDNA Synthesis SuperMix kit (Tiangen Biotech, Beijing, China) by following the manufacturer’s protocol. Real-time quantitative PCR (qRT-PCR) was performed using an Applied Biosystems (CA, USA) 7500 real-time PCR machine programmed at 95°C for 3 min, 39 cycles of 95°C for 15 s, 60°C for 30 s, and 72°C for 30 s, with a two-step qPCR kit (PrimeScriptTMRT Reagent Kit and SYBR Premix ExTaqTM, TaKaRa, Japan). The *Ib*actin gene was selected as the reference gene (Wang H. et al., 2016), with the targeting primers as sense: F 5´-CTGGTGTTATGGTTGGGATGG- 3´; antisense: 5´-GGGGTGCCTCGGTAAGAAG- 3´. Primers of the other genes are listed in **Supplementary Table S1**. Gene expression was calculated by following the 2^-ΔΔCt^ method afterward (Livak and Schmittgen, 2001). Each biological replicate was followed by four technical replicates.

### Transcriptomic and metabolomic analysis

The total RNA of the sweet potato roots was extracted as described above, then delivered to the Novogene Technology Co., Ltd. (Beijing, China) for RNA-seq and bioinformatic analysis as described in Zhu et al. (Zhu et al., 2018). The freeze-dried root samples were delivered to Metware Biotechnology Co., Ltd. (Wuhan, China) for metabolite isolation and analysis by the widely targeted metabolome (Chen et al., 2013; Yang et al., 2019). The details were as follows: The freeze-dried roots were crushed using a mixer mill (MM 400, Retsch) with a zirconia bead for 1.5 min at 30 Hz. 100mg powder was weighted and extracted overnight at 4°C with 0.6 ml 70% aqueous methanol. Following centrifugation at 10, 000g for 10 min, the extracts were absorbed (CNWBOND Carbon-GCB SPE Cartridge, 250mg, 3ml; ANPEL, Shanghai, China, www.anpel.com.cn/cnw) and filtrated (SCAA-104, 0.22μm pore size; ANPEL, Shanghai, China, http://www.anpel.com.cn/) before UPLC-MS/MS analysis. The sample extracts were analyzed using a UPLC-ESI-MS/MS system (UPLC, Shim-pack UFLC SHIMADZU CBM30A system, www.shimadzu.com.cn/; MS, Applied Biosystems 4500 Q TRAP, www.appliedbiosystems.com.cn/). The analytical conditions were as follows, UPLC: column, Waters ACQUITY UPLC HSS T3 C18 (1.8 µm, 2.1 mm*100 mm). The mobile phase consisted of solvent A, pure water with 0.04% acetic acid, and solvent B, acetonitrile with 0.04% acetic acid. Sample measurements were performed with a gradient program that employed the starting conditions of 95% A and 5% B. Within 10min, a linear gradient to 5% A and 95% B was programmed, and a composition of 5% A and 95% B was kept for 1min. Subsequently, a composition of 95% A and 5.0% B was adjusted within 0.10 min and kept for 2.9 min. The column oven was set to 40°C; the injection volume was 4μl. The effluent was alternatively connected to an ESI-triple quadrupole-linear ion trap (QTRAP)-MS. LIT and triple quadrupole (QQQ) scans were acquired on a triple quadrupole-linear ion trap mass spectrometer (Q TRAP), API 4500 Q TRAP UPLC/MS/MS System, equipped with an ESI Turbo Ion-Spray interface, operating in positive and negative ion mode and controlled by Analyst 1.6.3 software (AB Sciex). The ESI source operation parameters were as follows: ion source, turbo spray; source temperature 550°C; ion spray voltage (IS) 5500 V (positive ion mode)/-4500 V (negative ion mode); ion source gas I (GSI), gas II (GSII), and curtain gas (CUR) were set at 50, 60, and 30.0 psi, respectively; the collision gas (CAD) was high. Instrument tuning and mass calibration were performed with 10 and 100 μmol/L polypropylene glycol solutions in QQQ and LIT modes, respectively. QQQ scans were acquired as MRM experiments with collision gas (nitrogen) set to 5 psi. DP and CE for individual MRM transitions were done with further DP and CE optimization. A specific set of MRM transitions were monitored for each period according to the metabolites eluted within this period.

### Statistical analysis

Excel 2019, SPSS 26.0, and TBtools 1.0 software were utilized for the statistical analysis. A variety of analytical methods including the ANOVA, Duncan’s multiple range test, Pearson correlation coefficient, and Principal component analysis (PCA) were applied to reveal the statistical significance, P <0.05 was regarded as significant. The network visualization for the transcriptome and metabolomic data was performed using the Cytoscape 3.9.1 software (Shannon et al., 2003) by following the methods described online (http://www.cytoscape.org), which was also reported in a similar study on potatoes (Cho et al., 2016).

## Results

### Phenotypic differentiation in terms of the pigment distribution and accumulation in the fleshes of the four experimental sweet potato materials

The material 1143-1 showed white flesh while HS was orange-fleshed. By comparison, the other two sweet potato materials, DZ88 and DZ54, both had purple fleshes but were much darker in DZ54. The contents of β-carotene, total anthocyanins (TA), total flavonoids (TF), total phenolic acids (TP), and chlorogenic acid in the sweet potato fleshes were measured. Consistent with the flesh color phenotypes, the accumulation of β-carotene in HS was the highest (5.99 mg/100g fresh weight, FW) whereas DZ54 and DZ88 had almost 3-fold reductions (**Figure 1B**). The production of anthocyanin in the two purple sweet potato materials was the highest, peaking at 4.85 mg/g dried weight (DW) in DZ54 but showed a lesser amount in DZ88 (1.09 mg/g DW) (**Figure 1F**). As a result, levels of TF were correspondingly concordant with the anthocyanin accumulation trend in the sweet potato fleshes, except that 1143-1 demonstrated similar content (0.65 mg/g DW) relative to HS (**Figure 1E**). Intriguingly, the biosynthesis of chlorogenic acid in DZ88 was 1.6-fold higher than that in DZ54, and more than 2-fold that in 1143-1 and HS, but still followed the phenolic compound accumulation pattern among the four experimental materials (**Figure 1D**). The pigment distribution in the white flesh material 1143-1 remained at the minimum.

### Metabolomic characterization of the identifiable metabolites in sweet potato fleshes

**Figure 2:**
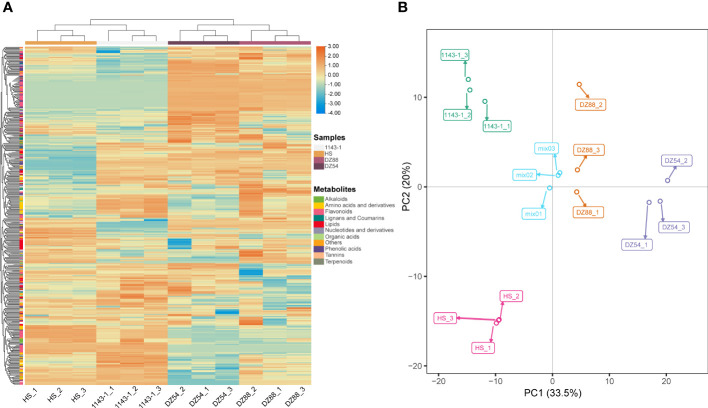
Overview of the total 418 metabolites distribution in the four experimental sweet potato materials. **(A)** Heat map visualization. The complete linkage hierarchical cluster analysis (HCA) method was applied to reflect the normalized metabolite content in each sample, which is represented by a single row with ranging color chroma from orange (high abundance) to blue (low abundance). **(B)** Two-dimensional principal component analysis (PCA) plot. The discrepancy and consistency among different experimental materials as well as each biological replicate of a sample were verified through the PCA.

A total of 418 metabolites (98 flavonoids, 77 phenolic acids, 51 lipids, 48 amino acids and their derivatives, 31 nucleotides and derivatives, 22 organic acids, 19 alkaloids, 11 lignans, and coumarins) have been detected in the metabolomic analysis (**Supplementary Table S2**). The distribution pattern of various metabolites ranging from alkaloids to terpenoids showed discrepant accumulation tendency in the four experimental sweet potato materials (**Figure 2A**). Phenolic compounds, such as flavonoid and phenolic acid, as well as other high carbon organics (i.e. lipid), were more preferred by the purple sweet potatoes DZ54 and DZ88, whereas other substances, such as organic acids, tannins, and lignans, were relatively more enriched in 1143-1 and HS. The PCA result reflected that the four materials could be generally divided into four independent groups (**Figure 2B**), with two principal components as PC1 (33.5%) and PC2 (20.0%), respectively, indicating that each sample could be characteristically distinguished from each other.

The flavonoid and phenolic substances related to the pigment metabolism in sweet potato flesh were thereafter specified (**Table 1**). In particular, a series of flavonoids and their derivatives, including anthocyanin (13 kinds), quercetin (16 kinds), kaempferol (5 kinds), and luteolin (4 kinds), as well as nine chlorogenic acids, were found to be differentially accumulated as the dominant metabolite fluxes affecting the pigmentation in the sweet potato root fleshes among the four experimental materials. Not surprisingly, the enrichment of anthocyanin in sweet potatoes is consistent with the root flesh colors. Purple sweet potatoes showed multiple folds of enhancement when compared to the non-purple materials, not only in the total anthocyanin contents but also in some specific components. For instance, DZ54 exhibited an almost 21-fold increase of cyanidin 3-feruloyl-p-hydroxybenzoylsophorside-5-glucoside relative to the 1143-1. The peonidin derivatives, such as peonidin 3-caffeoyl-p-hydroxybenzoyl-sophoroside-5-glucoside, presented a distinct difference between the two purple sweet potatoes (3.25-fold decrease in DZ88). However, regarding flavonoid branching, the two purple sweet potato materials presented an opposite trend compared to anthocyanin. The drastically reduced quercetin, kaempferol, and luteolin derivatives featured such differences. It was also interesting to find that the chlorogenic acids, particularly the maleoyl-caffeoylquinic acid, were more abundantly synthesized in purple sweet potatoes than in the non-purple ones. This may suggest a potential correlation between chlorogenic acid and anthocyanin accumulation.

**Table 1:** Differentially accumulated metabolites among the four experimental sweet potato materials.

Metabolite names	Log_2_ (Fold Change)					
	DZ54 vs 1143-1	DZ88 vs 1143-1	DZ54 vs HS	DZ88 vs HS	DZ54 vs DZ88	HS vs 1143-1
Anthocyanin (13)						
Cyanidin 3-(6′′-caffeoylsophoroside)-5-glucoside	13.52	13.05	13.52	13.05	–	–
Cyanidin 3-caffeoyl-p-hydroxybenzoylsophoroside-5-glucoside	18.58	17.25	18.58	17.25	1.33	–
Cyanidin 3-feruloyl-p-hydroxybenzoylsophorside-5-glucoside	20.92	17.75	20.92	17.75	3.17	–
Cyanidin 3-feruloylsophorside-5-glucoside	12.86	13.69	12.86	13.69	–	–
Cyanidin 3-p-hydroxybenzoylsophoroside-5-glucoside	15.91	15.49	15.91	15.49	–	–
Cyanidin-3-(sinapoyl)diglucoside-5-xyloside	12.82	13.58	12.82	13.58	–	–
Peonidin	-2.17	-4.97	–	–	2.80	-1.57
Peonidin-3-caffeoyl-p-hydroxybenzoyl-sophoroside-5-glucoside	20.39	17.13	20.39	17.13	3.25	–
Peonidin 3-feruloylsophoroside-5-glucoside	14.53	12.74	14.53	12.74	1.79	–
Peonidin-3-(6′-p-hydroxybenzoyl)-sophoroside-5-glucoside	14.63	12.82	14.63	12.82	1.81	–
Pelargonidin 3-caffeoyl-p-hydroxybenzoylsophorside-5-glucoside	15.23	12.78	15.23	12.78	2.46	–
Petunidin 3-O-glucoside	13.64	11.98	13.64	11.98	1.66	–
Malvidin	1.86	*-*	2.49	1.11	1.38	–
Quercetin derivatives (16)						
Quercetin 3-alpha-L-arabinofuranoside (Avicularin)	-9.32	-9.32	-12.19	-12.19	–	2.87
Quercetin 3-O-glucoside(Isotrifoliin)	-2.99	-2.01	-4.19	-3.20	–	1.19
Quercetin 3-O-β-D-xylopyranoside	–	–	-11.71	-11.71	–	11.71
Quercetin O-acetylhexoside	-11.51	-2.12	-13.65	-4.27	-9.39	4.75
Quercetin-3-O-α-L-rhamnopyranoside	-19.38	-19.38	-17.33	-17.33	–	-2.06
Quercetinn 3-O-(6′′-O-malonyl)-galactoside	-12.45	-12.45	-14.05	-14.05	–	1.60
Quercetin-O-glucoside	-3.36	-2.51	-4.46	-3.61	–	–
methylQuercetin O-hexoside	1.96	–	-2.79	–	–	4.75
Isoquercitrin	-3.47	-2.36	-4.54	-3.43	-1.10	–
Isorhamnetin-O-gallate	–	-1.10	–	–	1.10	–
Isorhamnetin O-acetyl-hexoside	–	–	-3.29	–	-2.68	–
Isorhamnetin-3-O-β-D-glucoside	2.44	2.40	-2.50	-2.54	–	4.94
Isorhamnetin 3-O-β-(2’’-O-acetyl-β-D-glucuronide)	-13.56	-13.56	-11.90	-11.90	–	-1.66
Hyperin	-3.46	-2.39	-4.37	-3.30	-1.07	1.27
Spiraeoside	-3.11	-2.20	-4.37	-3.46	–	1.24
Gossypitrin	-2.99	-2.17	-4.23	-3.41	–	–
Kaempferol derivatives (5)						
6-Hydroxykaempferol-7-O-glucoside	-2.78	-2.25	-4.08	-3.55	–	1.30
Astragalin	-7.59	-5.94	–	-3.26	–	-2.68
Kaempferol 3-O-(6′′-O-malonyl)-galactoside	-12.88	-12.88	-11.78	-11.78	–	–
Kaempferol 7-O-glucosdie	-18.74	-18.74	-16.25	-16.25	–	-2.49
Trifolin	-18.99	-6.27	-16.20	-3.48	-12.72	-2.79
Luteolin derivatives (4)						
Luteolin-4’-O-β-D-glucoside	-19.65	-19.65	-17.63	-17.63	–	-2.03
Luteolin-7-O-glucoside	-20.92	-5.36	-18.53	-2.97	-15.56	-2.39
Luteolin 3’-O-β-D-glucoside	-19.50	-19.50	-17.67	-17.67	–	-1.84
6-Hydroxyluteolin 5-glucoside	-2.94	-2.13	-4.17	-3.36	–	1.23
Chlorogenic acids (9)						
1-O-Caffeoylquinic acid (1-Caffeoylquinic acid)	2.22	1.35	1.99	1.12	–	–
4-O-Caffeoylquinic acid (Cryptochlorogenic acid)	2.04	–	1.16	–	1.05	–
5-O-Caffeoylquinic acid (Neochlorogenic acid)	1.92	1.16	1.75	–	–	–
3,5-di-O-Caffeoylquinic acid (Isochlorogenic acid A)	2.00	1.08	–	–	–	1.02
3,4-di-O-caffeoylquinic acid (Isochlorogenic acid B)	1.99	–	1.09	–	1.21	–
4,5-di-O-caffeoylquinic acid (Isochlorogenic acid C)	–	1.02	–	–	–	–
Trihydroxycinnamoylquinic acid	–	–	–	1.27	–	-1.10
Maleoyl-caffeoylquinic acid	1.89	1.41	14.96	14.48	–	-13.08
3-O-*p*-Coumaroyl quinic acid	2.48	2.31	2.17	2.00	–	–

Differentially accumulated metabolites were identified by threshold VIP (variable importance in projection) ≥1, and fold change was revealed via the log_2_ normalization. ‘-’ indicates that the values in the relevant comparison group were not significantly different from each other.

### Transcriptome analysis

**Figure 3:**
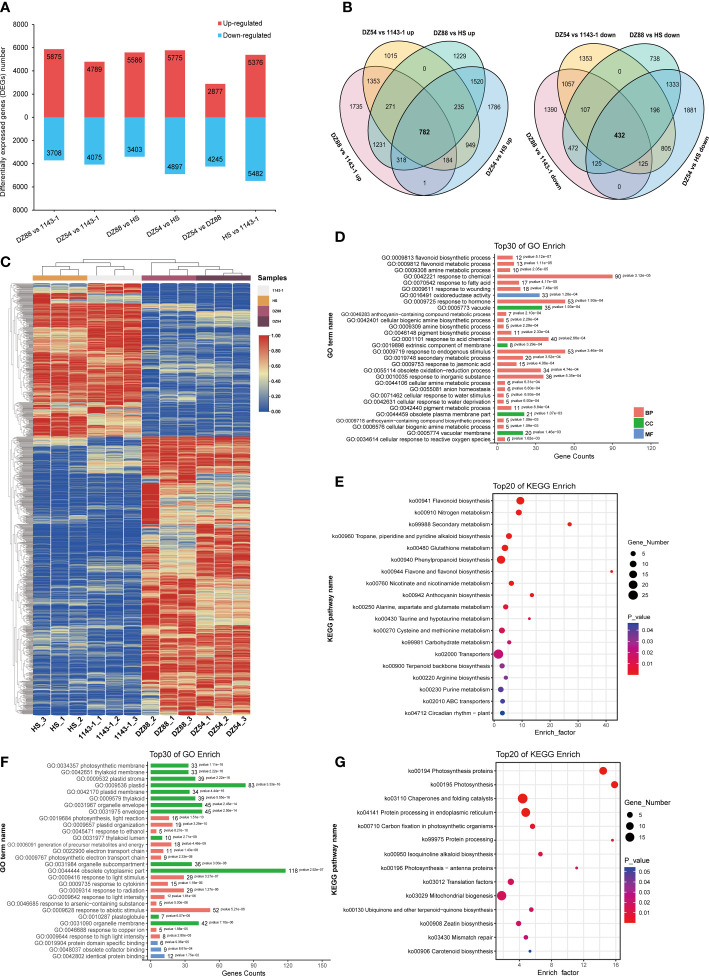
Analysis of differentially expressed genes (DEGs) derived from the comparative transcriptomics among the four experimental sweet potato materials. **(A)** Up/down-regulated DEG numbers. **(B)** Venn diagram display of the up/down-regulated DEGs. Four comparison groups were focused to reveal the anthocyanin accumulation differences: DZ54 vs 1143-1, DZ88 vs 1143-1, DZ54 vs HS, and DZ88 vs HS. **(C)** Heat map visualization of the expression levels of the DEGs. Colors ranging from red (high expression level) to blue (low expression level) represent the FPKM values. **(D)** Top 30 classifications of the upregulated DEGs *via* the GO enrichment. **(E)** Top 20 classifications of the upregulated DEGs *via* the KEGG enrichment. **(F)** Top 30 classifications of the downregulated DEGs *via* the GO enrichment. **(G)** Top 20 classifications of the downregulated DEGs *via* the KEGG enrichment.

A total of 50,893 genes have been identified in the RNAseq. Over 84.51% clean reads could be functionally annotated and 79.68% (**Supplementary Table S3**) were mapped uniquely against the Ipomoea batatas genome dataset(Yang et al., 2019). Six comparison groups, 1143-1 vs DZ88, 1143-1 vs DZ54, HS vs DZ88, HS vs DZ54, DZ88 vs DZ54, and 1143-1 vs HS, were divided to elucidate the gene expression differences among the four experimental sweet potatoes (**Figure 3A**). However, in order to investigate the differentially expressed genes (DEGs) specifically contributing to the anthocyanin biosynthesis, four pairwise comparison groups aimed to separately contrast the purple (DZ54 & DZ88) and non-purple (1143-1 & HS) materials were chosen. There are 1214 mutual DEGs screened from the four selected comparison groups, of which 782 were upregulated and 432 downregulated (**Figure 3B**) (**Supplementary Table S4**). The expression levels of the screened 1214 DEGs recorded as the FPKM value were reflected in the heat map (**Figure 3C**), it was distinct that a polarized expression trend appeared between the purple and non-purple groups, resulting in the highly regulated DEG expressions.

The gene ontology (GO) and Kyoto encyclopedia of genes and genomes (KEGG) enrichment analysis were simultaneously applied to categorize the DEGs. The top 30 of GO analysis revealed that a certain part of the 782 upregulated DEGs was enriched in the flavonoid metabolic process and anthocyanin-containing compound metabolic process (**Figure 3D**), whereas the plastidial, organelle, and cytoplasmic processes were mostly enriched with the 432 downregulated DEGs (**Figure 3F**). Likewise, the top 20 KEGG enrichment showed a similar pathway classification. Flavonoid biosynthesis, flavone and flavonol biosynthesis, and anthocyanin biosynthesis occupied a certain percentage of the upregulated DEGs (**Figure 3E**), with the downregulated pathways preferably focused on the protein processing and catalysis, and partially enriched in other pigments (i.e. carotenoid) and organic macromolecule (e.g. terpenoid, isoquinoline alkaloid) biosynthesis (**Figure 3G**). Such an interrelationship between the gene expression regulation and the potential pathway competition suggests that the anthocyanin biosynthesis in the purple sweet potato materials was not only attributed to a suite of specific genes, but also the highly regulated metabolite flux allocation and modification on the cellular level.

### Highly differentiated anthocyanin biosynthetic network implies the orchestrated gene expression regulation and metabolite flux redistribution

**Figure 4:**
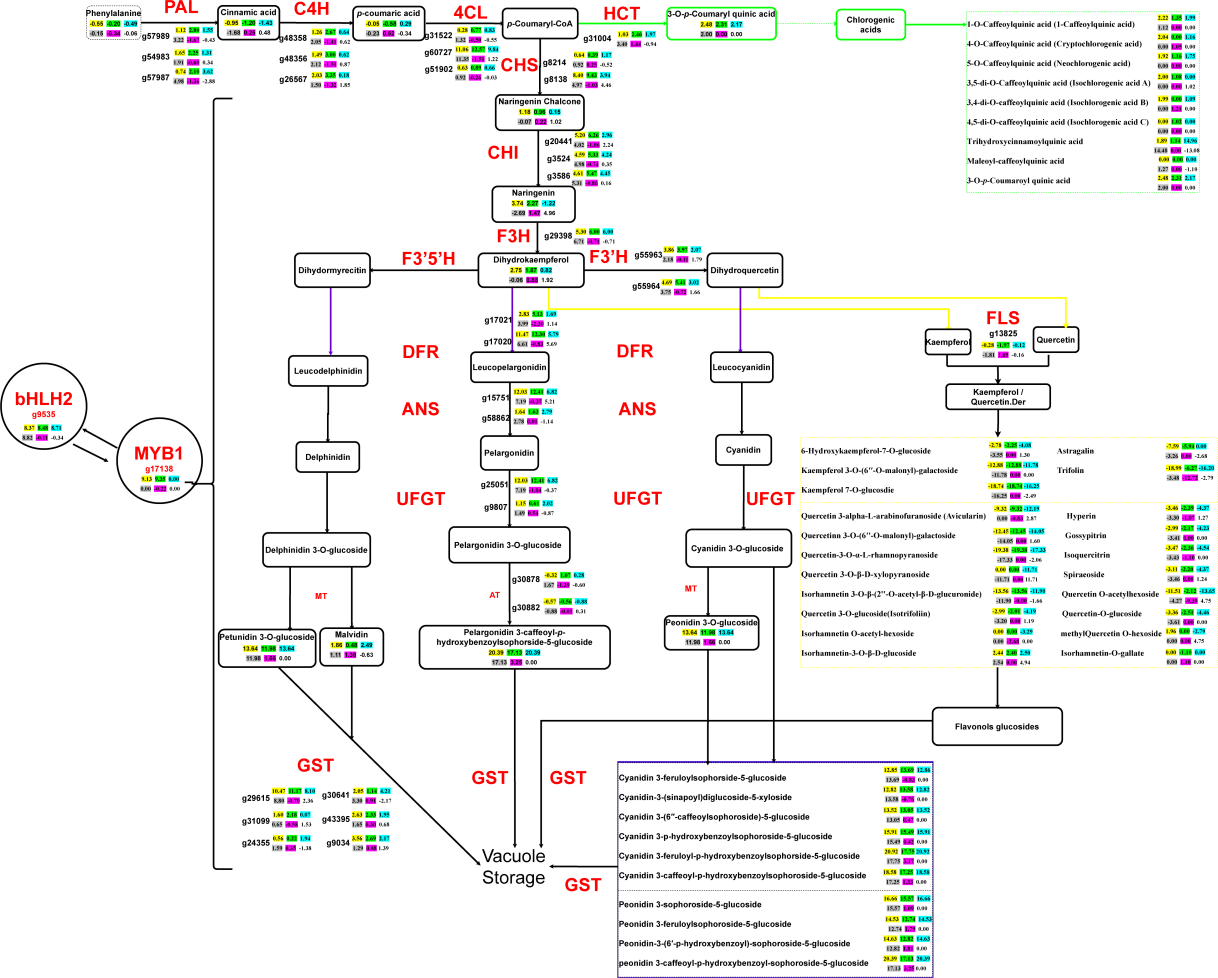
Central metabolic network dominating the gene expression and metabolite fluxion differences in the overall flavonoid biosynthesis of the four sweet potato materials. The pairwise comparison among the four sweet potato materials is divided into six groups, of which the yellow background represents DZ54 vs 1143-1, the green background represents DZ88 vs 1143-1, the blue background represents DZ54 vs HS, the grey background represents DZ88 vs HS, the purple background represents DZ54 vs DZ88, and the blank background represents HS vs 1143-1. Key regulatory genes catalyzing each enzymatic step are marked in red color, whose expression differences in the six pairwise comparison groups are reflected by the calibrated FPKM comparison levels of each unigene. The positive values indicate that the unigene expression was upregulated whereas the negative values mean the downregulated expression, which is the same as the metabolite abundance comparison of the products on the crucial metabolic nodes (values in the box). Important derivatives of the chlorogenic acid, quercetin, and kaempferol (flavonoid) as well as the anthocyanin biosynthetic pathways are collectively integrated into the three dashed boxes marked as green, yellow, and purple, respectively. *PAL*, phenylalanine ammonialyase; *C4H*, cinnamic acid 4-hydroxylase; *4CL*,4-coumarate CoA ligase; *CHS*, chalcone synthase; *CHI*, chalcone isomerase; *F3H*, flavanone 3-hydroxylase; *F3′H*, flavanoid 3′-hydroxylase; *F3´5´H*, flavonoid 3´,5´-hydroxylase; *DFR*, dihydroflavonol 4-reductase; *ANS*, anthocyanidin synthase/leucocyanidin oxygenase; *UFGT*, anthocyanidin 3-O-glucosyltransferase; *FLS*, flavonol synthesis; *HCT*, shikimate O-hydroxycinnamoyltransferase; *MT*, methylferase; *AT*, anthocyanidin 3-O-glucoside 6’’-O-acyltransferase; *GST*, glutathione S-transferase, *MYB1*, v-myb avian myeloblastosis viral oncogene homolog 1; *bHLH2*, basic Helix-Loop-Helix 2.

The joint transcriptomic and metabolomic analysis characterizing the regulatory network of anthocyanin biosynthesis in the four experimental sweet potato materials revealed the specific metabolic nodes that possibly led to the pigmentation differentiation (**Figure 4**). The significantly upregulated expression of the two transcriptional factors *bHLH2* (g9535) and *MYB1* (g17138), as well as the downstream structural genes, such as *4CL* (g60727), *CHS* (g8138), and *CHI* (g20441), which were involved in the initial catalytic steps of the anthocyanin production in the two PFSPs, had already determined the overall orientation towards which the substrates would be subsequentially converted. However, the biosynthetic pathways of other flavonoid components, mainly kaempferol and quercetin, also played a crucially competitive role in this respect. In particular, the branching of kaempferol and quercetin from the dihydrokaempferol and dihydroquercetin, under the catalysis of the *FLS* gene (g13825), was found to be the core factor leading to the differentiated pigmentation of the four sweet potato materials. Both of the two PFSPs demonstrated decreased expression levels of the *FLS* compared to the other two non-purple sweet potatoes, which had thereafter reduced the abundance of the relevant flavonoid derivatives.

Such a generally consistent tendency with the phenotypic performance (**Figure 3**) has thereby manifested that the substrate concentration remains another critical factor impacting the anthocyanin productivity, which largely corresponded to the enzymatic catalysis efficacy as a result of the gene expressions. For example, the abundance of naringenin in the PFSPs was approximately 2-fold higher than that in the colorless sweet potato. In line with the elevated expression level of the *F3H* gene (g29398), the subsequent conversion to dihydrokaempferol as a branching point that resulted in the diversified derivatization of anthocyanin compounds afterward, accordingly maintained a similar pattern. Likewise, the bioproduction of chlorogenic acids, which was diverted from the p-Counaryl-CoA through the catalysis of the *HCT* gene (g31004) with the maximum 3.4-fold increased expression level in DZ88 relative to HS, displayed significantly enhanced accumulation in the PFSPs (**Figure 3E**), while the relatively lower expression level of the *HCT* in HS when compared to 1143-1 did not exhibit any significant difference in both OFSP and WFSP. Despite the abundance of p-Counaryl-CoA was not detected, it still could be reflected that the abundant accumulation of chlorogenic acids in the PFSPs was controlled by the intercorrelated gene expression and substrate concentration, which regulate the distribution of metabolic fluxes together.

Other than that, the dramatically enhanced expression of the *GST* gene (g29615) in the PFSPs, which function as the key regulator in the vacuolar storage of anthocyanins, also suggested that the pigmentation of the four sweet potato materials could not only be attributed to the sequential substrate conversion efficiency but also the ultimate transportation of the final products into the designated organelles.

### Correlation analysis between the key metabolites and transcripts in the anthocyanin central metabolic network

**Figure 5:**
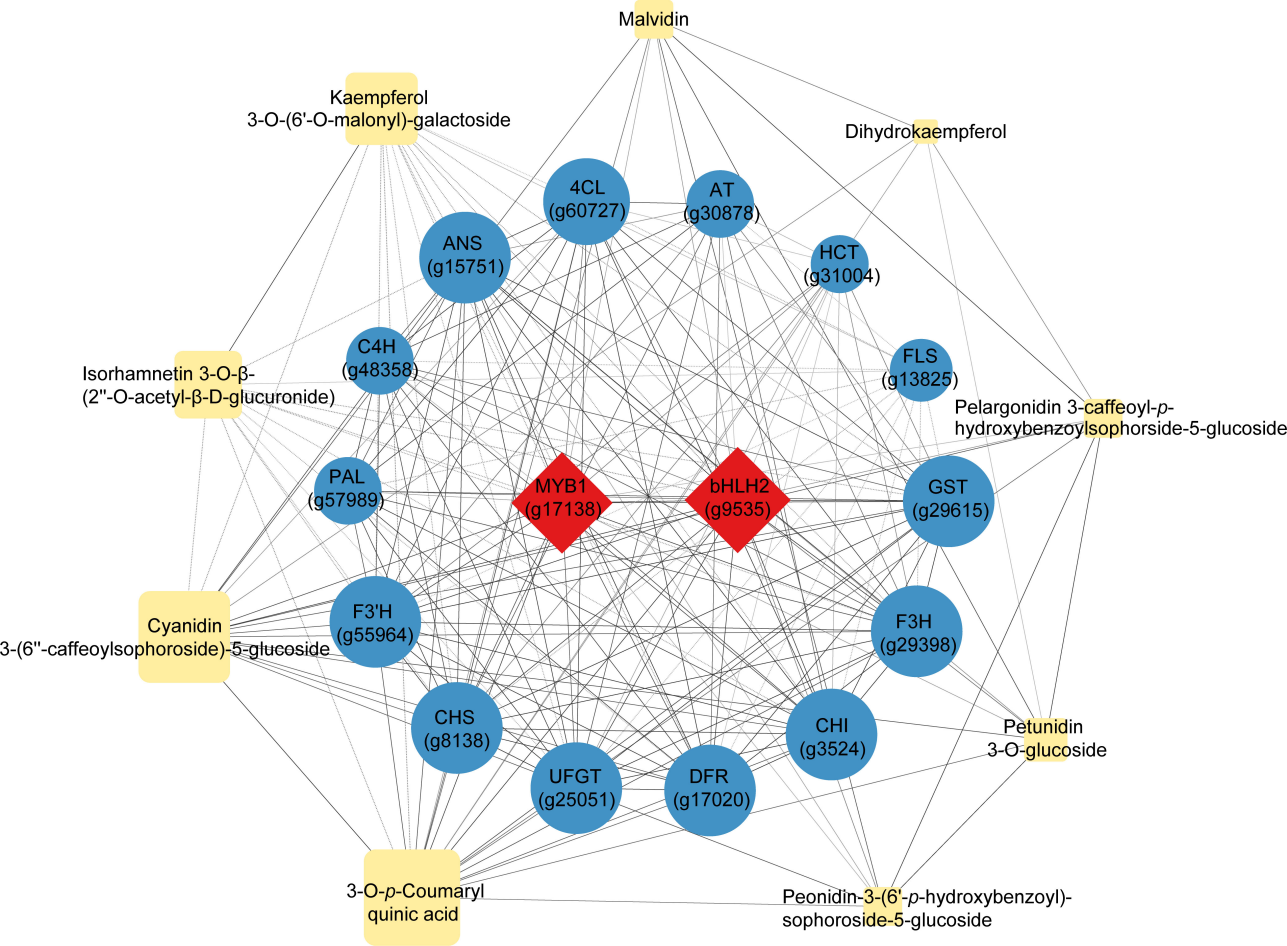
Correlation analysis of the nine representative differentially accumulated metabolites and 16 key differentially expressed regulatory genes enriched in the central anthocyanin metabolic network. Red rhombuses represent the transcriptional factors, blue circles represent the structural genes, and yellow rhomboids represent the metabolites. The larger the size of the graphics, the higher the relative expression or accumulation levels were. Full lines indicate the positive correlation whereas the dotted lines indicate the negative correlation (*p*<0.05), of which the coefficient absolute values were all above 0.5.

Nine representative differentially accumulated metabolites that reflected the most significant differences between the purple and non-purple sweet potato groups, including six anthocyanin derivatives, two quercetin and kaempferol derivatives, and dihydrokaempferol (**Table 1**), were selected for the correlation analysis with the 16 key regulatory genes dominating the anthocyanin biosynthesis (**Figure 5**) (**Supplementary Table S5**). The two transcriptional factors *MYB1* and *bHLH2* not only had an intimate correlation coefficient (0.939) reciprocally but showed highly orchestrated interactions with the other 14 downstream structural genes. Likewise, the gene crosslinking among the 14 structural genes was also distinct. For instance, the *GST* gene regulating the final step of the vacuolar anthocyanin storage had up to 0.969, 0.967, and 0.957 correlation coefficients with the *DFR*, *ANS*, and *UFGT*, respectively, which are the three genes responsible for the catalytic conversion and modification of anthocyanin end-products, while its correlation with the further upstream genes such as *PAL* (0.853) was relatively lower by contrast.

In terms of the correlation between gene regulation and metabolite formation, it was presented that the gene expression had a more straightforward effect on metabolite production as well as on metabolite interactions. In particular, *FLS* was found to be the gene with an exclusive function in controlling the biosynthesis of the two flavonoid products, Isorhamnetin 3-O-β-(2’’-O-acetyl-β-D-glucuronide) (0.577) and Kaempferol 3-O-(6′′-O-malonyl)-galactoside (0.599), hence its correlation with other anthocyanin products could not be revealed due to the pathway specificity. Conversely, other metabolites enriched in the anthocyanin class showed different levels of correlation with each other and the relevant genes, regardless of the gene expression distance or metabolite distribution in the whole pathway. Therefore, it could be certified that the overall regulation and interaction of the anthocyanin biosynthesis were highly targeted on both the gene-gene, gene-metabolite, and metabolite-metabolite dimensions. Other pathways differentiated from this mechanism, i.e., chlorogenic acid (*HCT*) and flavonoid (*FLS*), were relatively independent of such correlative patterns.

### qRT-PCR analysis of the key regulatory genes enriched in the anthocyanin biosynthetic network

**Figure 6:**
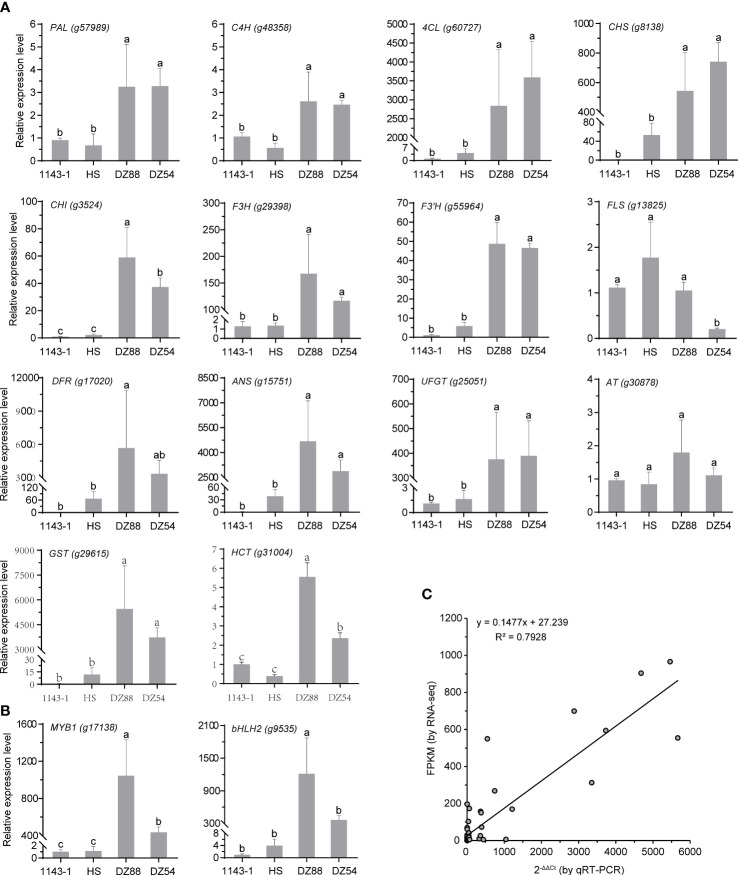
Relative expressions of the 16 key regulatory genes enriched in the central metabolic network of anthocyanin biosynthesis in sweet potatoes. The *Ibactin* gene was used as the reference gene. The data represent the mean values ± SD of three biological replicates. The letters above the bars are based on ANOVA, bars marked with different letters are statistically significantly different at P<0.05. **(A)** Structural genes. **(B)** Transcriptional factors. **(C)** Correlation of gene expression results obtained from qRT-PCR analysis and RNA-Seq for colour-related genes in four sweet potato materials.

The 16 key structural genes enriched in the central metabolic network controlling the anthocyanin biosynthesis were further validated through the qRT-PCR analysis, including the two critical transcriptional factors *MYB1* and *bHLH2* (**Figure 6**). Generally, the gene expression levels were consistent with the transcriptomic data (R^2^>0.79) as well as the phenotypic performance. With several-fold (e.g. *PAL*, *C4H*) to thousands-fold (e.g. *4CL*, *DFR*, *ANS*, *GST*) upregulation of the gene expressions, the metabolic fluxes were substantially oriented to the anthocyanin anabolism in the PFSPs. However, the expression of the *FLS* was found to be downregulated in the PFSPs, especially in DZ54 but had the highest expression level in HS. This may partially explain why the abundance of kaempferol and quercetin derivatives in the HS material was multiple-fold higher than that in the two purple sweet potato materials (**Table 1**).

## Discussion

Colorful sweet potato is rich in secondary metabolites, which are the dominant factors leading to the pigmentation in root flesh (Zhao et al., 2022). Anthocyanin belongs to the flavonoid compound that can present a purple or red color in plant tissue, whereas the high accumulation of β-carotene results in an orange color (Hu et al., 2011; Liu et al., 2017). In this study, we have conducted a joint omics analysis by applying the comparative transcriptomic and metabolomic methodology to reveal the coloring mechanism differences among the four sweet potato materials 1143-1 (white flesh), HS (orange flesh), DZ88, and DZ54 (purple flesh). There were 38 differentially accumulated pigment metabolites (**Table 1**) and 1214 DEGs (**Figure 3A**) identified from the six pairwise comparison groups of these four colored materials. This result was consistent with previous reports that the composition of phenolic compounds in white, yellow, blue, and black naked barley was very different, with the black sample containing the most types of compounds and a higher content of phenolic acid and flavonoids(Ge et al., 2021). Focusing on the anthocyanin metabolic differentiation, it has been recognized that glycosylated cyanidins and peonidins with caffeoyl, feruloyl, p-hydroxybenzoyl, and sinapoyl residue modifications were the primarily stable pigment components in purple sweet potatoes compared to other components (Truong et al., 2010). It was found that16 genes with significantly different transcriptional levels were enriched as the key regulators impacting the central anthocyanin biosynthesis in sweet potatoes (**Figure 5**). This is in agreement with the study reported by Li et al. (Li et al., 2021).

Other than the anthocyanin, it was intriguing to discover that chlorogenic acids were abundantly accumulated in purple sweet potatoes (**Figure 1D**), particularly in the DZ88 material (1.15 mg/g DW), which was almost 2-fold higher than what was observed in 1143-1 and HS. Nine differentially accumulated categories of chlorogenic acids were identified in the metabolomic analysis. Of which the maleoyl-caffeoylquinic acid displayed up to a 15-fold increase in the purple materials (**Table 1**), implying an interrelated but independent relationship with the metabolic conversions between pigments and chlorogenic acids (Valiñas et al., 2017). In specific, the biosynthesis of chlorogenic acids initially originated from the *p*-Coumaryl-CoA, which is an intermediate substrate localized on the upstream catalytic steps of the general phenylpropanoid biosynthetic pathway **(** **Figure 4** **).** Despite this potential competition, both the white and orange-fleshed sweet potatoes had a certain level of chlorogenic acids accumulation (**Figure 1D**), while the dramatically elevated expressions of the *HCT* gene in purple sweet potatoes contributed to the abundance of chlorogenic acids biosynthesis (**Figure 6**). Nevertheless, even though the accumulation of anthocyanin was coordinated by a suite of highly regulated structural genes (Zhao et al., 2022), such pathway differentiation did not seem to significantly affect anthocyanin biosynthesis. We thereby hypothesized that the redistribution of the *p*-Coumaryl-CoA into either the anthocyanin or chlorogenic acid biosynthetic pathways in sweet potato could be a sophisticatedly harmonized catalytic step in adjusting the reaction rate of the anthocyanin production efficacy (Valiñas et al., 2017). However, it remains to be further investigated.

As illustrated in **Figure 3**, the competition or redistribution of dihydrokaempferol and dihydroquercetin, between the downstream production of anthocyanin products and the derivatization of flavonoid compounds (*i.e.* kaempferol and quercetin), was also reflected to be a critical metabolic node at which the purple and non-purple sweet potatoes were distinguished in the central anthocyanin biosynthesis. The upregulated expression of the *FLS* gene in HS, which had a higher accumulation of kaempferol and quercetin derivatives relative to the PFSWs (**Table 1**), could be the major contributor to such differentiation. Davies et al. raised a similar theory concerning pigmentation in petunia (*Petunia hybrida*) (Davies et al., 2003). It was shown that the expression of the *FLS* gene had directly led to the production of colorless flavonols, while the colored anthocyanin could be significantly synthesized when the *FLS* was downregulated by the antisense RNA technology. It was the same as observed in the study with *Muscari armeniacum* flowers, where the upregulation of the *FLS* gene inhibited the *DFR* expression to a certain extent and induced the whitening phenotype (Lou et al., 2014). This negative correlation between the *FLS* and *DFR* genes, as well as the two intermediate substrates dihydrokaempferol and dihydroquercetin, were also reflected in the sweet potatoes (**Figure 5**). Hence, the reallocation of intermediate substrates, which was largely determined by the expression of specific genes (*e.g. FLS*), demonstrated that the transcriptional discrepancy in the gene expressions and the gene-metabolite interactions might have played a crucial role in the metabolite flux repartitioning, which thereafter caused the distinct pigmentary performances in the purple and non-purple sweet potatoes (Zhang et al., 2022).

Our study showed that the two transcriptional factors *MYB1* and *bHLH2* not only had significantly upregulated expression in the two PFSPs but had highly orchestrated interactions with the other 14 downstream structural genes. However, we didn’t detect the expression of *WD40*. In plants, the MYB either functions individually or can bind to the bHLH and *WD40* to form an MBW complex to induce the expressions of the downstream structural genes. Previous studies showed that the expression level of *WD40* genes, for instance, eggplant *SmWD40*, pepper *CaWD40*, and potato *StAN11*, were comparable between anthocyanin-pigmented and non-pigmented tissues (Stommel et al., 2009; Liu et al., 2015; Stommel and Dumm, 2015). Their expression levels hardly changed with altered transcript levels of structural genes or anthocyanin content (Stommel and Dumm, 2015). At present, the decisive role of *WD40* protein in regulating anthocyanin synthesis is still unclear, and the structural characteristics, functional properties, interaction mechanism with other factors, and target DNA binding sequence of the transcriptional regulatory complex need to be further studied.

In addition, because of the activation of *MYB1* and *bHLH2*, the expression of structural genes, such as *DFR*, was upregulated, and therefore the expression of the *FLS* gene decreased. Although DZ54 has more anthocyanins, the expression levels of its structural genes were lower than that of DZ88. This was probably due to the specific variation in the expression levels of these structural genes through various and complex regulation mechanisms resulting in quantitative and qualitative variations of anthocyanins, underlying the difference of colorations observed between species, genotypes, organs, or even between various positions on the same plant tissue (Qiao et al., 2019).

By contrast, other secondary metabolites, such as the total phenols and β-carotene in the four experimental materials, demonstrated a generally consistent fluctuation tendency in analogy with other sweet potato germplasms, for example, the Turkey cultivars Beniazuma, Koganesengan, and Kotobuki (Dincer et al., 2011), which seems to be a conservative mechanism in colorful and colorless sweet potatoes (Wang et al., 2018). Nevertheless, the significantly differentiated central anthocyanin metabolic network between the purple and non-purple sweet potato materials still sheds light on the anthocyanin component divergence. In our study, 14 categories of anthocyanin, including one glycosylated pelargonidin, one glycosylated petunidin, one malvidin, five glycosylated peonidin, and six glycosylated cyanidin, were recognized, whereas there were only seven or eight kinds identified in a previous study (Wang A. et al., 2018; Zhang et al., 2022). Despite the technical difficulty in accurately verifying the monomer of anthocyanin (Li et al., 2012), we still detected more glycosylated derivatives in the purple sweet potatoes DZ88 and DZ54 (**Table 1**), which all displayed higher accumulation than the others. The non-target metabolomics has served as a useful tool in the rapid authentication of anthocyanin substances in the non-photosynthetic tissue of plants (Chen et al., 2013), which also explained to a larger extent the coloring mechanism, for example, the dark hue in purple sweet potato.

Compared to other anthocyanin-rich plants, such as black hawk raspberry (Gansch et al., 2009), black waxy rice, and grape (Khanal et al., 2010; Sutharut and Sudarat, 2012), the anthocyanin content of purple sweet potato may not be that considerable in terms of the industrial concern (Huang et al., 2006). However, advantages, such as the much lower production cost, higher yield, and better environmental adaptability relative to tree plants, have made the colorful sweet potatoes a more economical and broader source for commercial anthocyanin production (Odake et al., 1994; Chen et al., 2019; Bennett et al., 2021). In this regard, the two purple sweet potato materials DZ54 and DZ88 may have more potential in both scientific research and industrial development. Besides the component divergence of anthocyanin in these two materials, the higher chlorogenic acid content as an important natural antioxidant would also bring more application values, not just as experimental material but as targeted breeding candidates (Torres et al., 2019).

To conclude, the white (1143-1), orange (HS), and purple-fleshed (DZ54 and DZ88) sweet potatoes diversified but conserved regulatory mechanisms in the secondary metabolism. The competition of intermediate substrates in the central anthocyanin metabolic network under the regulation of several specific genes was found to be the major reason why such differentiation appeared (Davies et al., 2003; Lou et al., 2014; Valiñas et al., 2017). Our data obtained from the joint omics analysis has provided useful information for the further understanding of the molecular mechanisms underlying the pigmentation difference in colorful sweet potatoes.

## Data availability statement

The original contributions presented in the study are publicly available. This data can be found here: NCBI, PRJNA881010, PRJNA881014, PRJNA881013, and PRJNA881012.

## Author contributions

JX designed the research, performed the experiments, analyzed the data, and wrote the manuscript. XX improved the manuscript. ML and XW were material growers and visualized data. HG was the material provider, and conceived and designed the project. All authors have read and agreed to the published version of the manuscript.

## Supplementary material

Supplementary Table 1 Primers used in quantitative reverse transcription polymerase chain reaction (qRT-PCR) in this study.

Supplementary Table 2 Information of the total 418 metabolites detected in the four experimental sweet potato materials through the metabolomic analysis.

Supplementary Table 3 Summary of the RNA-seq results for validation of the raw data validity.

Supplementary Table 4 Detailed message of 1214 differentially expressed genes (782 upregulated and 432 downregulated) identified from the comparative transcriptomics.

Supplementary Table 5 Values of the correlation coefficient reflecting the gene-metabolite, metabolite-metabolite, gene-gene, and metabolite-gene interactions visualized in **Figure 5** (P<0.05).

## References

[B1] Azeem M. Mu T. H. Zhang M. (2020). Effects of high hydrostatic pressureand soaking solution on proximate composition, polyphenols, anthocyanins, β-carotene, and antioxidant activity of white, orange, and purple fleshed sweet potato flour. Food Sci. Technol. Int. 26 (5), 388–402. doi: 10.1177/1082013219892716 31870191

[B2] Bennett A. A. Mahood E. H. Fan K. Moghe G. D. (2021). Untargeted metabolomics of purple and orange-fleshed sweet potatoes reveals a large structural diversity of anthocyanins and flavonoids. Sci. Rep. 11 (1), 16408. doi: 10.1038/s41598-021-95901-y 34385537 PMC8361111

[B3] Bulgakov V. P. Avramenko T. V. Tsitsiashvili G. S. (2017). Critical analysis of protein signaling networks involved in the regulation of plant secondary metabolism: focus on anthocyanins. Crit. Rev. Biotechnol. 37 (6), 685–700. doi: 10.3109/07388551.2016.1141391 26912350

[B4] Chen W. Gong L. Guo Z. Wang W. Zhang H. Liu X.. (2013). A novel integrated method for Large-scale detection, identification, and quantification of widely targeted metabolites: Application in the study of rice metabolomics. Mol. Plant 6 (6), 1769–1780. doi: 10.1093/mp/sst080 23702596

[B5] Chen C. C. Lin C. Chen M. H. Chiang P. Y. (2019). Stability and quality of anthocyanin in purple sweet potato extracts. Foods. 8 (9), 393. doi: 10.3390/foods8090393 31489943 PMC6770014

[B6] Cho K. Cho K. S. Sohn H. B. Ha I. J. Hong S. Y. Lee H.. (2016). Network analysis of the metabolome and transcriptome reveals novel regulation of potato pigmentation. J. Exp. Bot. 67 (5), 1519–1533. doi: 10.1093/jxb/erv549 26733692 PMC4762390

[B7] Davies K. M. Schwinn K. E. Deroles S. C. Manson D. G. Lewis D. H. Bloor S. J.. (2003). Enhancing anthocyanin production by altering competition for substrate between flavonol synthase and dihydroflavonol 4-reductase. Euphytica. 131, 259–268. doi: 10.1023/A:1024018729349

[B8] Dincer C. Karaoglan M. Erden F. Tetik N. Topuz A. Ozdemir F. (2011). Effects of baking and boiling on the nutritional and antioxidant properties of sweet potato [Ipomoea batatas (L.) lam.] cultivars. Plant Foods Hum. Nutr. 66 (4), 341–347. doi: 10.1007/s11130-011-0262-0 22101780

[B9] Dixon R. A. Liu C. Jun J. H. (2013). Metabolic engineering of anthocyanins and condensed tannins in plants. Curr. Opin. Biotechnol. 24, 329–335. doi: 10.1016/j.copbio.2012.07.004 22901316

[B10] Do Q. D. Angkawijaya A. E. Tran-Nguyen P. L. Huynh L. H. Soetaredjo F. E. Ismadji S.. (2014). Effect of extraction solvent on total phenol content, total flavonoids content, and antioxidant activity of limnophila aromatic. J. Food Drug Analysis. 22 (3), 296–302. doi: 10.1016/j.jfda.2013.11.001 PMC9354875 28911418

[B11] FAO (Food and Agriculture Organization) (2013). Food and agricultural commodities production (Rome: FAO).

[B12] Gansch H. Weber C. A. Lee C. Y. (2009). Antioxidant capacity and phenolic phytochemicals in black raspberries. New York Fruit quarterly. 17, 20–23. doi: 10.21273/HORTSCI.43.7.2039

[B13] Ge X. Jing L. Zhao K. Su C. Zhang B. Zhang Q.. (2021). The phenolic compounds profile, quantitative analysis and antioxidant activity of four naked barley grains with different color. Food Chem. 335, 127655. doi: 10.1016/j.foodchem.2020.127655 32731125

[B14] Hatier J. H. B. Gould K. S. (2018). Anthocyanin function in vegetative organs. In: Winefield C. Davies K. Gould K. (eds). Anthocyanins (New York, NY: Springer). doi: 10.1007/978-0-387-77335-3_1

[B15] He Q. Lu Q. He Y. Wang Y. Zhang N. Zhao W.. (2020). Dynamic changes of the anthocyanin biosynthesis mechanism during the development of heading Chinese cabbage (Brassica rapa l.) and arabidopsis under the control of BrMYB2. Front. Plant Sci. 11. doi: 10.3389/fpls.2020.593766 PMC7785979 33424889

[B16] He W. Zeng M. Chen J. Jiao Y. Niu F. Tao G.. (2016). Identification and quantitation of anthocyanins in purple-fleshed sweet potatoes cultivated in china by UPLC-PDA and UPLC-QTOF-MS/MS. J. Agric. Food Chem. 64, 171–177. doi: 10.1021/acs.jafc.5b04878 26687974

[B17] Hichri I. Heppel S. C. Pillet J. Léon C. Czemmel S. Delrot S.. (2010). The basic helix-loop-helix transcription factor MYC1 is involved in the regulation of the flavonoid biosynthesis pathway in grapevine. Mol. Plant 3 (3), 509–523. doi: 10.1093/mp/ssp118 20118183

[B18] Huang Y.-C. Chang Y.-H. Shao Y.-Y. (2006). Effects of genotype and treatment on the antioxidant activity of sweet potato in Taiwan. Food Chem. 98, 529–538. doi: 10.1016/j.foodchem.2005.05.083

[B19] Hu Y. Deng L. Chen J. Zhou S. Liu S. Fu Y.. (2016). An analytical pipeline to compare and characterise the anthocyanin antioxidant activities of purple sweet potato cultivars. Food Chem. 194, 46–54. doi: 10.1016/j.foodchem.2015.07.133 26471525

[B20] Hu C. Gong Y. Jin S. Zhu Q. (2011). Molecular analysis of a UDP-glucose: Flavonoid 3-o-glucosyltransferase (UFGT) gene from purple potato (Solanum tuberosum). Mol. Biol. Rep. 38, 561–567. doi: 10.1007/s11033-010-0141-z 20358295

[B21] Jiang T. Zhou J. Liu W. Tao W. He J. Jin W.. (2020). The anti-inflammatory potential of protein-bound anthocyanin compounds from purple sweet potato in LPS-induced RAW264.7 macrophages. Food Res. Int. 137, 109647. doi: 10.1016/j.foodres.2020.109647 33233226

[B22] Khanal R. C. Howard L. R. Prior R. L. (2010). Effect of heating on the stability of grape and blueberry pomace procyanidins and total anthocyanins. Food Res. Int. 43, 1464–1469. doi: 10.1016/j.foodres.2010.04.018

[B23] Kim H. W. Kim J. B. Cho S. M. Chung M. N. Lee Y. M. Chu S. M.. (2012). Anthocyanin changes in the Korean purple-fleshed sweet potato, shinzami, as affected by steaming and baking. Food Chem. 130 (4), 966–972. doi: 10.1016/j.foodchem.2011.08.031

[B24] Lee J. Durst R. W. Wrolstad R. E. (2005). Determination of total monomeric anthocyanin pigment content of fruit juices, natural colorants, and wines by the pH differential method: collaborative study. J. AOAC Int. 88, 1269–1278. doi: 10.1093/jaoac/88.5.1269 16385975

[B25] Li H. Deng Z. Zhu H. Hu C. Liu R. Young J. C.. (2012). Highly pigmented vegetables: Anthocyanin compositions and their role in antioxidant activities. Food Res. Int. 46 (1), 250–259. doi: 10.1016/j.foodres.2011.12.014

[B26] Li Q. Kou M. Li C. Zhang Y. G. (2021). Comparative transcriptome analysis reveals candidate genes involved in anthocyanin biosynthesis in sweetpotato (Ipomoea batatas l.). Plant Physiol. Biochem. 158, 508–517. doi: 10.1016/j.plaphy.2020.11.035 33272792

[B27] Li G. Lin Z. Zhang H. Liu Z. Xu Y. Xu G.. (2019). Anthocyanin accumulation in the leaves of the purple sweet potato (Ipomoea batatas l.) cultivars. Molecules. 24 (20), 3743. doi: 10.3390/molecules24203743 31627373 PMC6832942

[B28] Li P. Li Y. Zhang F. Zhang G. Jiang X. Yu H.. (2016). The arabidopsis UDP- glycosyltransferases UGT79B2 and UGT79B3, contribute to cold, salt and drought stress tolerance *via* modulating anthocyanin accumulation. Plant J. 89, 85–103. doi: 10.1111/tpj.13324 27599367

[B29] Lim S. Xu J. Kim J. Chen T. Y. Su X. Standard J.. (2013). Role of anthocyanin-enriched purple-fleshed sweet potato p40 in colorectal cancer prevention. Mol. Nutr. Food Res. 57 (11), 1908–1917. doi: 10.1002/mnfr.201300040 23784800 PMC3980565

[B30] Liu Y. Lin-Wang K. Deng C. Warran B. Wang L. Yu B.. (2015). Comparative transcriptome analysis of white and purple potato to identify genes involved in anthocyanin biosynthesis. PloS One 10, e0129148. doi: 10.1371/journal.pone.0129148 26053878 PMC4459980

[B31] Liu X. Xiang M. Fan Y. Yang C. Zeng L. Zhang Q.. (2017). A root-preferential DFR-like gene encoding dihydrokaempferol reductase involved in anthocyanin biosynthesis of purple-fleshed sweet potato. Front. Plant Sci. 8. doi: 10.3389/fpls.2017.00279 PMC5329058 28293252

[B32] Livak K. J. Schmittgen T. D. (2001). Analysis of relative gene expression data using real-time quantitative PCR and the 2 ΔΔ c T method. Methods. 25 (4), 402–408. doi: 10.1006/meth.2001.1262 11846609

[B33] Lou Q. Liu Y. Qi Y. Jiao S. Tian F. Jiang L.. (2014). Transcriptome sequencing and metabolite analysis reveals the role of delphinidin metabolism in flower colour in grape hyacinth. J. Exp. Botany. 65, 3157–3164. doi: 10.1093/jxb/eru168 24790110 PMC4071837

[B34] Low J. W. Arimond M. Osman N. Cunguara B. Zano F. Tschirley D. (2007). A food-based approach introducing orange-fleshed sweet potatoes increased vitamin a intake and serum retinol concentrations in young children in rural Mozambique1–3. J. Nutr. 137, 1320–1327. doi: 10.1093/jn/137.5.1320 17449599

[B35] Mano H. Ogasawara F. Sato K. Higo H. Minobe Y. (2007). Isolation of a regulatory gene of anthocyanin biosynthesis in tuberous roots of purple-fleshed sweet potato. Plant Physiol. 143, 1252–1268. doi: 10.1104/pp.106.094425 17208956 PMC1820918

[B36] Meng F. L. Guo H. C. (2019). Effect analysis of anti-UV-B enhancement of two sweet potato cultivars. Chin. J. Agrometeorol. 40 (05), 29–36. doi: 10.3969/j.issn.1000-6362

[B37] Mitra S. (2012). Nutritional status of orange-fleshed sweet potatoes in alleviating vitamin a malnutrition through food based approach. J. Nutr. Food Sci. 2, 160. doi: 10.4172/2155-9600.1000160

[B38] Nurdjanah S. Nurdin S. U. Astuti S. Manik V. E. (2022). Chemical components, antioxidant activity, and glycemic response values of purple sweet potato products. Int. J. Food Sci. 4, 7708172. doi: 10.1155/2022/7708172 PMC9033360 35465219

[B39] Odake K. Hatanaka A. Kajiwara T. Muroi T. Nishiyama K. Yamakawa O.. (1994). Evaluation method and breeding of purple sweet potato “YAMAGAWA MURASAKI” (Ipomoea batatas POIR.) for raw material of food colorants. Nippon Shokuhin Kagaku Kogaku Kaishi. 41, 287–293. doi: 10.3136/nskkk1962.41.287

[B40] Qiao Z. Liu S. Zeng H. Li Y. Wang X. Chen Y.. (2019). Exploring the molecular mechanism underlying the stable purple-red leaf phenotype in lagerstroemiaindica cv. ebony embers. Int. J. Mol. Sci. 20 (22), 5636. doi: 10.3390/ijms20225636 31718025 PMC6888693

[B41] Ruttarattanamongkol K. Chittrakorn S. Weerawatanakorn M. Dangpium N. (2016). Effect of drying conditions on properties, pigments and antioxidant activity retentions of pretreated orange and purple-fleshed sweet potato flours. J. Food Sci. Technol. 53 (4), 1811–1822. doi: 10.1007/s13197-015-2086-7 27413208 PMC4926894

[B42] Shannon P. Markiel A. Ozier O. Baliga N. S. Wang J. T. Ramage D.. (2003). Cytoscape: a software environment for integrated models of biomolecular interaction networks. Genome Res. 13 (11), 2498–2504. doi: 10.1101/gr.1239303 14597658 PMC403769

[B43] Stommel J. R. Dumm J. M. (2015). Coordinated regulation of biosynthetic and regulatory genes coincides with anthocyanin accumulation in developing eggplant fruit. J. Am. Soc Hortic. Sci. 140, 129–135. doi: 10.21273/JASHS.140.2.129

[B44] Stommel J. R. Lightbourn G. J. Winkel B. S. Griesbach R. J. (2009). Transcription factor families regulate the anthocyanin biosynthetic pathway in capsicum annuum. J. Am. Soc Hortic. Sci. 134, 244–251. doi: 10.21273/JASHS.134.2.244

[B45] Sun C. Deng L. Du M. Zhao J. Chen Q. Huang T.. (2020). A transcriptional network promotes anthocyanin biosynthesis in tomato flesh. Mol. Plant 13, 42–58. doi: 10.1016/j.molp.2019.10.010 31678614

[B46] Sun H. Mu T. Liu X. Zhang M. Chen J. (2014). Purple sweet potato (Ipomoea batatas l.) anthocyanins: preventive effect on acute and subacute alcoholic liver damage and dealcoholic effect. J. Agric. Food Chem. 62 (11), 2364–2373. doi: 10.1021/jf405032f 24564852

[B47] Sun H. Zhang P. Zhu Y. Lou Q. He S. (2018). Antioxidant and prebiotic activity of five peonidin-based anthocyanins extracted from purple sweet potato (Ipomoea batatas (L.) lam.). Sci. Rep. 8 (1), 5018. doi: 10.1038/s41598-018-23397-0 29568082 PMC5864876

[B48] Sutharut J. Sudarat J. (2012). Total anthocyanin content and antioxidant activity of germinated colored rice. Int. Food Res. 19, 215–221.

[B49] Tang Y. Cai W. Xu B. (2015). Profiles of phenolics, carotenoids and antioxidative capacities of thermal processed white, yellow, orange and purple sweet potatoes grown in guilin, China. Food Sci. Hum. Wellness. 4, 123–132. doi: 10.1016/j.fshw.2015.07.003

[B50] Torres A. Basurto F. Navarro-Ocana A. (2019). Quantitative analysis of the biologically active compounds present in leaves of Mexican sweet potato accessions: Phenols, flavonoids, anthocyanins, 3,4,5-Tri-Caffeoylquinic acid and 4-Feruloyl-5-Caffeoylquinic acid. Plant Foods Hum. Nutr. 74 (4), 531–537. doi: 10.1007/s11130-019-00774-2 31713022

[B51] Truong V. D. Deighton N. Thompson R. T. McFeeters R. F. Dean L. O. Pecota K. V.. (2010). Characterization of anthocyanins and anthocyanidins in purple-fleshed sweetpotatoes by HPLC-DAD/ESI-MS/MS. J. Agric. Food Chem. 58, 404–410. doi: 10.1021/jf902799a 20017481

[B52] Tsushida T. Suzuki M. Kurogi M. (1994). Evaluation of antioxidant activity of vegetable extracts and determination of some active compounds. J. Japanese Soc. Food Sci. Technol. (Nippon Shokuhin Kogyo Gakkaishi). 41, 611–618. doi: 10.3136/nskkk1962.41.611

[B53] Tumuhimbise G. A. Namutebi A. Muyonga J. H. (2009). Microstructre and *in vitro* beta carotene bioaccessibilty of heat processed organe fleshed sweet potato. Plant Food Hum. Nutr. 64, 312–318. doi: 10.1007/s11130-009-0142-z 19908145

[B54] US Agency for International Development (2014). Orange-fleshed sweet potatoes: Improving lives in Uganda. Available at: https://reliefweb.int/report/uganda/orange-fleshed-sweet-potatoes-improving-lives-uganda [Accessed Jan 3, 2014]

[B55] Valiñas M. A. Lanteri M. L. Ten Have A. Andreu A. B. (2017). Chlorogenic acid, anthocyanin and flavan-3-ol biosynthesis in flesh and skin of Andean potato tubers (Solanum tuberosum subsp. andigena). Food Chem. 229, 837–846. doi: 10.1016/j.foodchem.2017.02.150 28372251

[B56] Wang H. Fan W. Wu Y. Zhang P. Wang C. Yang J.. (2018). A novel glycosyltransferase catalyses the transfer of glucose to glucosylated anthocyanins in purple sweetpotato. J. Exp. Bot. 69, 5444–5459. doi: 10.1093/jxb/ery305 30124996 PMC6255700

[B57] Wang S. Hong T. Jian W. Wei C. Liu X. Jie L.. (2016). Spatio-temporal distribution and natural variation of metabolites in citrus fruits. Food Chem. 199, 8–17. doi: 10.1016/j.foodchem.2015.11.113 26775938

[B58] Wang A. Li R. Ren L. Gao X. Zhang Y. Ma Z.. (2018). A comparative metabolomics study of flavonoids in sweet potato with different flesh colors (Ipomoea batatas (L.) lam). Food Chem. 260, 124–134. doi: 10.1016/j.foodchem.2018.03.125 29699652

[B59] Wang W. Li J. Wang Z. Gao H. Su L. Xie J.. (2014). Oral hepatoprotective ability evaluation of purple sweet potato anthocyanins on acute and chronic chemical liver injuries. Cell Biochem. Biophysics. 69 (3), 539–548. doi: 10.1007/s12013-014-9829-3 24442992

[B60] Wang H. Yang J. Zhang M. Fan W. Firon N. Pattanaik S.. (2016). Altered phenylpropanoid metabolism in the maize lc-expressed sweet potato (Ipomoea batatas) affects storage root development. Sci. Rep. 6 (1), 18645. doi: 10.1038/srep18645 26727353 PMC4698713

[B61] Waterhouse A. L. (2003). Determination of total phenolics. Curr. Protoc. Food Anal. Chem. 6, I1.1.1–I1.1.8.

[B62] Wei Q. H W. Mei L. Yuan B. Zeng M. M. Gao D. M.. (2019). Anthocyanin composition and storage degradation kinetics of anthocyanins-based natural food colourant from purple-fleshed sweet potato. Int. J. Food Sci. Technol 54 (8), 2529–2539. doi: 10.1111/ijfs.14163

[B63] Xie F. Burklew C. E. Yang Y. Liu M. Xiao P. Zhang B.. (2012). *De novo* sequencing and a comprehensive analysis of purple sweetpotato (Ipomoea batatas l.) transcriptome. Planta. 236, 101–113. doi: 10.1007/s00425-012-1591-4 22270559

[B64] Xu W. Dubos C. Lepiniec L. (2015). Transcriptional control of flavonoid biosynthesis by MYB-bHLH-WDR complexes. Trends Plant Sci. 20, 176–185. doi: 10.1016/j.tplants.2014.12.001 25577424

[B65] Yang M. Yang J. Su L. Sun K. Li D. Liu Y.. (2019). Metabolic profile analysis and identification of key metabolites during rice seed germination under low-temperature stress. Plant Sci. 289, 110282. doi: 10.1016/j.plantsci.2019.110282 31623771

[B66] Zhang Y. G. Fang B. P. (2006). Descriptors and data standard for sweet potato [Ipomoea batatas (L.) Lam. ] (Beijing: Pressed by China Agriculture Press Co., Ltd.), P89.

[B67] Zhang L. Hu J. Han X. Li J. Gao Y. Richards C. M.. (2019). A high-quality apple genome assembly reveals the association of a retrotransposon and red fruit colour. Nat. Commun. 10 (1), 1494. doi: 10.1038/s41467-019-09518-x 30940818 PMC6445120

[B68] Zhang R. Li M. Tang C. Jiang B. Yao Z. Mo X.. (2022). Combining metabolomics and transcriptomics to reveal the mechanism of coloration in purple and cream mutant of sweet potato (Ipomoea batatas l.). Front. Plant Sci. 13. doi: 10.3389/fpls.2022.877695 PMC9116297 35599902

[B69] Zhang Z. F. Lu J. Zheng Y. L. Wu D. M. Hu B. Shan Q.. (2013). Purple sweet potato color attenuates hepatic insulin resistance *via* blocking oxidative stress and endoplasmic reticulum stress in high-fat-diet-treated mice. J. Nutr. Biochem. 24 (6), 1008–1018. doi: 10.1016/j.jnutbio.2012.07.009 22995384

[B70] Zhao D. Zhao L. Liu Y. Zhang A. Xiao S. Dai X.. (2022). Metabolomic and transcriptomic analyses of the flavonoid biosynthetic pathway for the accumulation of anthocyanins and other flavonoids in sweetpotato root skin and leaf vein base. J. Agric. Food Chem. 70 (8), 2574–2588. doi: 10.1021/acs.jafc.1c05388 35175040

[B71] Zhi Q. Lei L. Li F. Zhao J. C. Yin R. Ming J. (2019). The anthocyanin extracts from purple-fleshed sweet potato exhibited anti-photoaging effects on ultraviolent b-irradiated BALB/c-nu mouse skin. J. Funct. Foods. 64, 103640. doi: 10.1016/j.jff.2019.103640

[B72] Zhu F. Cai Y. Z. Yang X. Ke J. Corke H. (2010). Anthocyanins, hydroxycinnamic acid derivatives, and antioxidant activity in roots of different Chinese purple-fleshed sweetpotato genotypes. J. Agric. Food Chem. 58 (13), 7588. doi: 10.1021/jf101867t 20524661

[B73] Zhu C. Li X. Zheng J. (2018). Transcriptome profiling using illumina- and SMRT-based RNAseq of hot pepper for in-depth understanding of genes involved in CMV infection. Gene. 666, 123–133. doi: 10.1016/j.gene.2018.05.004 29730427

